# Alectinib Versus Crizotinib in Asian Patients With Treatment-Naïve Advanced *ALK*-Positive NSCLC: Five-Year Update From the Phase 3 ALESIA Study

**DOI:** 10.1016/j.jtocrr.2024.100700

**Published:** 2024-06-27

**Authors:** Caicun Zhou, You Lu, Sang-We Kim, Thanyanan Reungwetwattana, Jianying Zhou, Yiping Zhang, Jianxing He, Jin-Ji Yang, Ying Cheng, Se-Hoon Lee, Jianhua Chang, Jian Fang, Zhe Liu, Lilian Bu, Li Qian, Tingting Xu, Venice Archer, Magalie Hilton, Mingzhu Zhou, Li Zhang

**Affiliations:** a Department of Medical Oncology, Shanghai Pulmonary Hospital, Tongji University, Shanghai, People's Republic of China; b Department of Thoracic Oncology, West China Hospital, Sichuan University, Chengdu, People's Republic of China; c Department of Oncology, Asan Medical Center, University of Ulsan College of Medicine, Seoul, South Korea; d Division of Medical Oncology, Department of Medicine, Faculty of Medicine Ramathibodi Hospital, Mahidol University, Bangkok, Thailand; e Department of Respiratory Diseases, The First Affiliated Hospital, Zhejiang University School of Medicine, Hangzhou, People's Republic of China; f Department of Thoracic Oncology, Zhejiang Cancer Hospital, Hangzhou, People's Republic of China; g Department of Thoracic Surgery, The First Affiliated Hospital of Guangzhou Medical University, Guangzhou, People's Republic of China; h Guangdong Lung Cancer Institute, Guangdong General Hospital, Guangzhou, People's Republic of China; i Department of Oncology, Jilin Cancer Hospital, Changchun, People's Republic of China; j Samsung Medical Center, Sungkyunkwan University School of Medicine, Seoul, South Korea; k Cancer Hospital and Shenzhen Hospital, Chinese Academy of Medical Sciences and Peking Union Medical College, Shenzhen, People's Republic of China; l Department of Thoracic Oncology, Beijing Cancer Hospital, Beijing, People's Republic of China; m Department of Oncology, Beijing Chest Hospital, Capital Medical University, Beijing, People's Republic of China; n Department of Data Science, Roche (China) Holding Ltd., Shanghai, People's Republic of China; o Department of Clinical Science, Roche (China) Holding Ltd., Shanghai, People's Republic of China; p Product Development, Roche Products Ltd., Welwyn Garden City, United Kingdom; q Product Development Data Sciences, F. Hoffmann-La Roche Ltd., Basel, Switzerland; r Department of Safety, Roche (China) Holding Ltd., Shanghai, People's Republic of China; s Sun Yat-sen University Cancer Center, State Key Laboratory of Oncology in South China, Collaborative Innovation Center for Cancer Medicine, Guangzhou, People's Republic of China

**Keywords:** ALESIA, Alectinib, *ALK*, Crizotinib, NSCLC

## Abstract

**Introduction:**

Previous results from the phase 3 ALESIA study (NCT02838420) revealed that alectinib (a central nervous system [CNS]-active, ALK inhibitor) had clinical benefits in treatment-naïve Asian patients with advanced *ALK*-positive NSCLC, consistent with the global ALEX study. We present updated data after more than or equal to 5 years of follow-up from the “last patient in” date.

**Methods:**

Adult patients with treatment-naïve, advanced *ALK*-positive NSCLC from mainland China, South Korea, and Thailand were randomized 2:1 to receive twice-daily 600 mg alectinib (n = 125) or 250 mg crizotinib (n = 62). The primary endpoint was investigator-assessed progression-free survival. Secondary or exploratory endpoints included overall survival, objective response rate, time to CNS progression, and safety.

**Results:**

At the data cutoff (May 16, 2022), the median survival follow-up was 61 and 51 months in the alectinib and crizotinib arms, respectively. Median progression-free survival was 41.6 months with alectinib versus 11.1 months with crizotinib (stratified hazard ratio = 0.33, 95% confidence interval: 0.23–0.49). Overall survival data remain immature; 5-year overall survival rates were 66.4% (alectinib arm) versus 56.1% (crizotinib arm). Objective response rate was 91.2% versus 77.4% with alectinib and crizotinib, respectively. CNS progression was delayed with alectinib versus crizotinib (cause-specific hazard ratio = 0.16, 95% confidence interval: 0.08–0.32). Median treatment duration was longer with alectinib versus crizotinib (42.3 versus 12.6 mo). No new safety signals were observed.

**Conclusions:**

With four additional years of follow-up, these updated results confirm the clinical benefit and manageable safety of alectinib in Asian patients with advanced *ALK*-positive NSCLC, and confirm alectinib as a standard-of-care treatment for patients with advanced *ALK*-positive NSCLC.

## Introduction

More than 50% of lung cancer cases worldwide are recorded in Asia, where 21% of cancer deaths occur owing to lung cancer.^1^ This highlights the need for improved treatment strategies in Asian patients. Alectinib is a highly selective, central nervous system (CNS)-active ALK inhibitor, which is recommended as a first-line treatment option for patients with advanced *ALK*-positive NSCLC in a number of treatment guidelines.^1^, ^2^, ^3^

Alectinib was approved for the treatment of *ALK*-positive NSCLC in Japan at a dose of 300 mg twice daily on the basis of results of the single-arm phase 1-2 AF-001JP study (JapicCTI-101264) in ALK inhibitor-naïve Japanese patients.^4^ Confirmatory data were provided by the randomized phase 3 J-ALEX study (JapicCTI-132316), which compared the efficacy and safety of 300 mg twice-daily alectinib with 250 mg twice-daily crizotinib in ALK inhibitor-naïve Japanese patients with *ALK*-positive NSCLC, who were chemotherapy naïve or who had received one previous chemotherapy regimen. Treatment crossover between arms was permitted following study drug discontinuation.^5^ Final progression-free survival (PFS) data from J-ALEX demonstrated superiority in independent review facility-assessed median PFS in patients who received alectinib (n = 103; 34.1 mo, 95% confidence interval [CI]: 22.1–not estimable [NE]) versus crizotinib (n = 104; 10.2 mo, 95% CI: 8.3–12.0) with a hazard ratio (HR) of 0.37 (95% CI: 0.26–0.52]).^6^ However, final overall survival (OS) data from J-ALEX did not reveal superiority of alectinib over crizotinib; median OS was not reached (NR) in either treatment arm (alectinib arm: 95% CI: 70.6–NE; crizotinib arm: 95% CI: 68.5–NE; HR = 1.03, 95% CI: 0.67–1.58), and the 5-year OS rate was 60.9% (95% CI: 51.4–70.3) with alectinib and 64.1% (95% CI: 54.9–73.4) with crizotinib.^7^

In the USA and Europe, alectinib was approved as a first-line treatment for patients with *ALK*-positive NSCLC on the basis of data from the global randomized phase 3 ALEX study (NCT02075840), which compared the efficacy and safety of alectinib at 600 mg versus crizotinib at 250 mg, twice daily, in treatment-naïve patients with advanced *ALK*-positive NSCLC.^8^ At the primary analysis of ALEX, investigator-assessed PFS was significantly longer in patients treated with alectinib versus crizotinib: median PFS was NE (95% CI: 17.7–NE) in the alectinib arm versus 11.1 months (95% CI: 9.1–13.1) in the crizotinib arm (HR = 0.47, 95% CI: 0.34–0.65, *p* < 0.001).^8^ Mature PFS data from ALEX confirmed a significant improvement in investigator-assessed median PFS for alectinib (34.8 mo, 95% CI: 17.7–NE) relative to crizotinib (10.9 mo, 95% CI: 9.1–12.9), with a HR of 0.43 (95% CI: 0.32–0.58).^9^ Of note, OS data from ALEX were immature at the updated analysis, with only 37% of patients having events (stratified HR = 0.67, 95% CI: 0.46–0.98); median OS was NE in the alectinib arm versus 57.4 months (95% CI: 34.6–NE) in the crizotinib arm. Despite this immaturity of the OS data, a clinically meaningful improvement in the 5-year OS rate was reported with alectinib (62.5%, 95% CI: 54.3–70.8) versus crizotinib (45.5%, 95% CI: 33.6–57.4).^9^ Finally, alectinib also revealed intracranial activity; a CNS response occurred in 17 out of 21 patients with CNS metastases in the alectinib arm (CNS response rate, 81%; 95% CI: 58–95), and in 11 out of 22 patients with CNS metastases in the crizotinib arm (CNS response rate, 50%; 95% CI: 28–72).^8^

In the randomized phase 3 ALESIA study (NCT02838420), treatment-naïve Asian patients with advanced *ALK*-positive NSCLC were enrolled to receive the globally approved 600 mg twice-daily alectinib dose; the primary objective was to assess whether the PFS benefit of alectinib in this patient population was consistent with that observed in the global ALEX study. In the primary analysis of ALESIA (data cutoff: May 31, 2018), comprising 125 patients in the alectinib arm and 62 patients in the crizotinib arm, the PFS benefit of alectinib versus crizotinib observed in ALEX was revealed in Asian patients with advanced *ALK*-positive NSCLC^10^: investigator-assessed PFS was significantly prolonged with alectinib versus crizotinib (median PFS NE [95% CI: 20.3–NE] versus 11.1 mo [95% CI: 9.1–13.0], respectively; HR = 0.22, 95% CI: 0.13–0.38, *p* < 0.0001). The favorable efficacy of alectinib was consistently observed across secondary endpoints, including independent review committee (IRC)-assessed time to CNS progression.^10^ Here, we report updated data from ALESIA (data cutoff: May 16, 2022), with a follow-up of more than or equal to 5 years from the date of the last patient in.

## Materials and Methods

### Study Design

Full study details have been described previously.^10^ Briefly, the intent-to-treat (ITT) population, defined as all randomized patients, comprised Asian patients aged more than or equal to 18 years with previously untreated, stage IIIB or IV *ALK*-positive NSCLC, who were enrolled from 21 sites in mainland China, South Korea, and Thailand. Eligibility criteria included an Eastern Cooperative Oncology Group performance status of 0 to 2, measurable baseline disease per Response Evaluation Criteria in Solid Tumors v1.1, and a life expectancy of more than or equal to 12 weeks. Patients with asymptomatic brain metastases were eligible; those with asymptomatic progressive disease (PD) in the CNS could receive localized treatment and continue with the study treatment until systemic PD or symptomatic PD in the CNS.

Patients were randomized 2:1 to receive either alectinib 600 mg orally or crizotinib 250 mg orally, twice daily, until PD, unacceptable toxicity, consent withdrawal, or death. Randomization was performed centrally by means of an interactive voice or web response system and was stratified by Eastern Cooperative Oncology Group performance status (0 or 1 versus 2) and baseline CNS metastases (yes versus no). Although treatment crossover between study arms was not permitted, patients could receive any available treatment after discontinuation from study treatment.

The study was conducted in accordance with the principles of the Declaration of Helsinki, the International Council for Harmonization guidelines for Good Clinical Practice, and country-specific laws and regulations. The study protocol was reviewed by the institutional review board. All patients provided written informed consent.

### Endpoints and Assessments

All patients had regular tumor imaging, including brain magnetic resonance imaging, at baseline and every 8 weeks until PD; responses were evaluated using Response Evaluation Criteria in Solid Tumors v1.1. The primary endpoint was investigator-assessed PFS. Secondary endpoints included IRC-assessed PFS, IRC-assessed time to CNS progression, objective response rate, duration of response, OS, and safety. Endpoints that were assessed by the IRC were only undertaken for the primary analysis; investigator-assessed time to CNS progression was included as an exploratory endpoint in this updated analysis instead. Adverse events (AEs) were graded using the National Cancer Institute Common Terminology Criteria for Adverse Events v4.0.

### Statistical Analyses

The primary objective of the ALESIA study was to determine whether the investigator-assessed PFS benefit with alectinib was consistent with the benefit reported in the global ALEX trial. Consistency was defined as maintaining greater than or equal to 50% of risk reduction from ALEX (i.e., as the HR for investigator-assessed PFS for ALEX was 0.47 [53% risk reduction], if the point estimate of the HR from ALESIA was less than 0.735, then the primary objective to determine consistency would be met).

Kaplan–Meier methodology was used to estimate time-to-event endpoints for each treatment arm with corresponding 95% CI, and a stratified Cox regression model using the stratification factors for randomization was used to estimate the HR and 95% CI of the treatment effect. The Clopper–Pearson method was used to estimate the proportion of patients who achieved an objective response with corresponding 95% CI, and proportions of responses were compared using a Mantel–Haenszel test on the basis of the stratification factors. Non-CNS progression without previous CNS progression, and death without previous CNS or non-CNS progression, were regarded as competing risks for CNS progression; therefore, HRs were calculated on the basis of cause-specific hazard functions to account for competing risks in the time to CNS progression analysis. The probability of CNS disease progression, non-CNS disease progression, and death were each estimated with the use of cumulative incidence functions. The *p* values presented for the efficacy endpoints are descriptive only.

## Results

### Patients

Baseline characteristics of the ITT population have been described previously.^10^ Briefly, between August 3, 2016, and May 16, 2017, 187 patients were randomized to receive alectinib (n = 125) or crizotinib (n = 62). Baseline patient characteristics of the ITT population were generally balanced between the treatment arms, as described previously (Supplementary Table 1).^10^ In total, IRC-assessed baseline CNS metastases were present in 44 patients in the alectinib arm and 23 patients in the crizotinib arm.

### Updated Efficacy Outcomes

In this updated analysis (data cutoff: May 16, 2022), the median duration of survival follow-up was 61 months for alectinib-treated patients and 51 months for crizotinib-treated patients. A lower proportion of patients had discontinued treatment with alectinib (76 of 125 [60.8%]) versus crizotinib (57 of 62 [91.9%]); most discontinuations were due to PD in both arms (58 of 125 [46.4%] versus 46 of 62 [74.2%], respectively).

**Figure 1:**
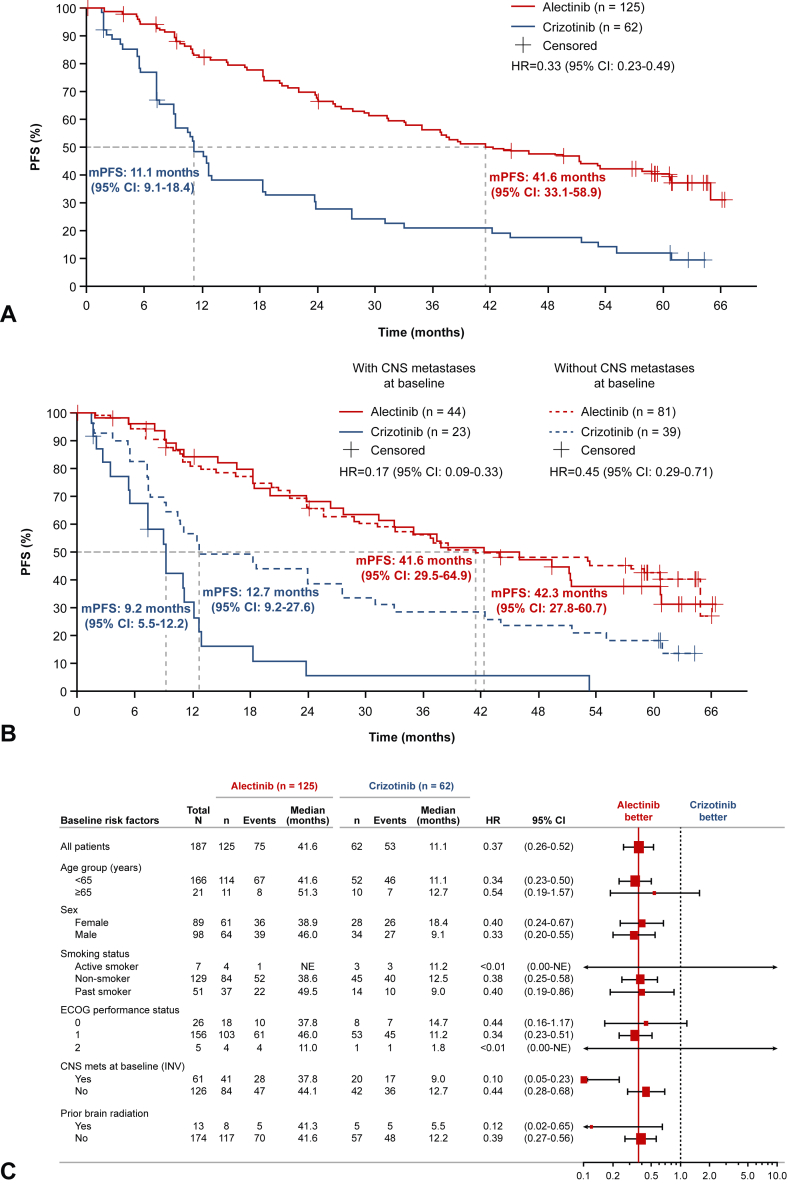
Updated investigator-assessed PFS *(A)* in the ITT population, *(B)* in patients with and without CNS metastases at baseline, and *(C)* in prespecified patient subgroups. CI, confidence interval; CNS, central nervous system; ECOG, Eastern Cooperative Oncology Group; HR, hazard ratio; INV, investigator; ITT, intent-to-treat; mPFS, median progression-free survival; PFS, progression-free survival.

In the ITT population, investigator-assessed PFS was prolonged in patients who received alectinib versus crizotinib (median PFS 41.6 mo [95% CI: 33.1–58.9] versus 11.1 mo [95% CI: 9.1–18.4], respectively; stratified HR = 0.33, 95% CI: 0.23–0.49, *p* < 0.0001; Fig. 1*A*). In patients with baseline CNS metastases, median PFS was 42.3 months (95% CI: 27.8–60.7) with alectinib versus 9.2 months with crizotinib (95% CI: 5.5–12.2) (HR = 0.17; 95% CI: 0.09–0.33), and in patients without baseline CNS metastases, median PFS was 41.6 months (95% CI: 29.5–64.9) versus 12.7 months (95% CI: 9.2–27.6) (HR = 0.45, 95% CI: 0.29–0.71), respectively (Fig. 1*B*). Improved PFS with alectinib versus crizotinib was also observed across several prespecified patient subgroups (Fig. 1*C*).

In the ITT population, the investigator-assessed objective response rate remained unchanged from the primary analysis,^10^ and was 91.2% (114 of 125, 95% CI: 84.8–95.5) in the alectinib arm versus 77.4% (48 of 62, 95% CI: 65.0–87.1) in the crizotinib arm. The median duration of response was longer in patients who received alectinib (44.3 mo, 95% CI: 33.2–58.9) versus crizotinib (9.4 mo, 95% CI: 7.4–17.0), with a stratified HR of 0.33 (95% CI: 0.22–0.50).

**Figure 2:**
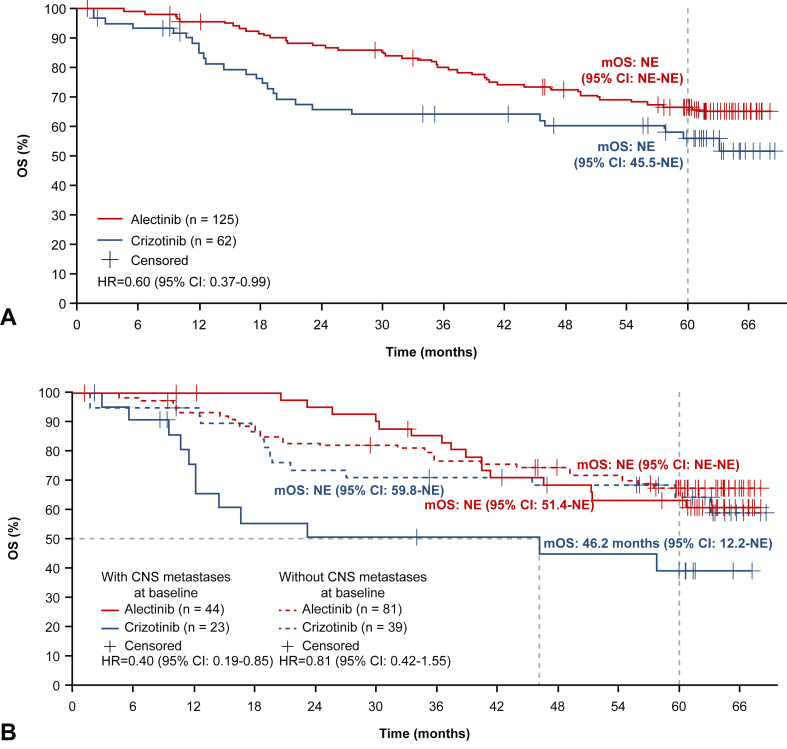
Updated OS *(A)* in the ITT population, and *(B)* in patients with and without CNS metastases at baseline. CI, confidence interval; CNS, central nervous system; HR, hazard ratio; ITT, intent-to-treat; mOS, median overall survival; NE, not estimable; OS, overall survival.

Overall survival data remain immature, with 32.8% (41 of 125) of patients in the alectinib arm and 41.9% (26 of 62) of patients in the crizotinib arm having had an event. An increase in the survival duration was seen in the alectinib versus the crizotinib arm in the ITT population (stratified HR = 0.60, 95% CI: 0.37–0.99); median OS was NE in the alectinib arm (95% CI: NE–NE) and in the crizotinib arm (95% CI: 45.5–NE) (Fig. 2*A*). The 5-year OS rate was 66.4% (95% CI: 57.9–74.9) with alectinib versus 56.1% (95% CI: 43.0–69.1) with crizotinib. In patients with baseline CNS metastases, median OS was NE (95% CI: 51.4–NE) in the alectinib arm versus 46.2 months (95% CI: 12.2–NE) in the crizotinib arm (HR = 0.40, 95% CI: 0.19–0.85), and the 5-year OS rate was 63.6% (95% CI: 48.9–78.3) versus 39.3% (95% CI: 17.4–61.2), respectively (Fig. 2B). In patients without baseline CNS metastases, median OS was NE in either treatment arm (alectinib arm: 95% CI: NE–NE; crizotinib arm: 95% CI: 59.8–NE) (HR = 0.81, 95% CI: 0.42–1.55), and the 5-year OS rate was 67.8% (95% CI: 57.4–78.2) with alectinib and 64.9% (95% CI: 49.3–80.4) with crizotinib (Fig. 2*B*).

**Figure 3:**
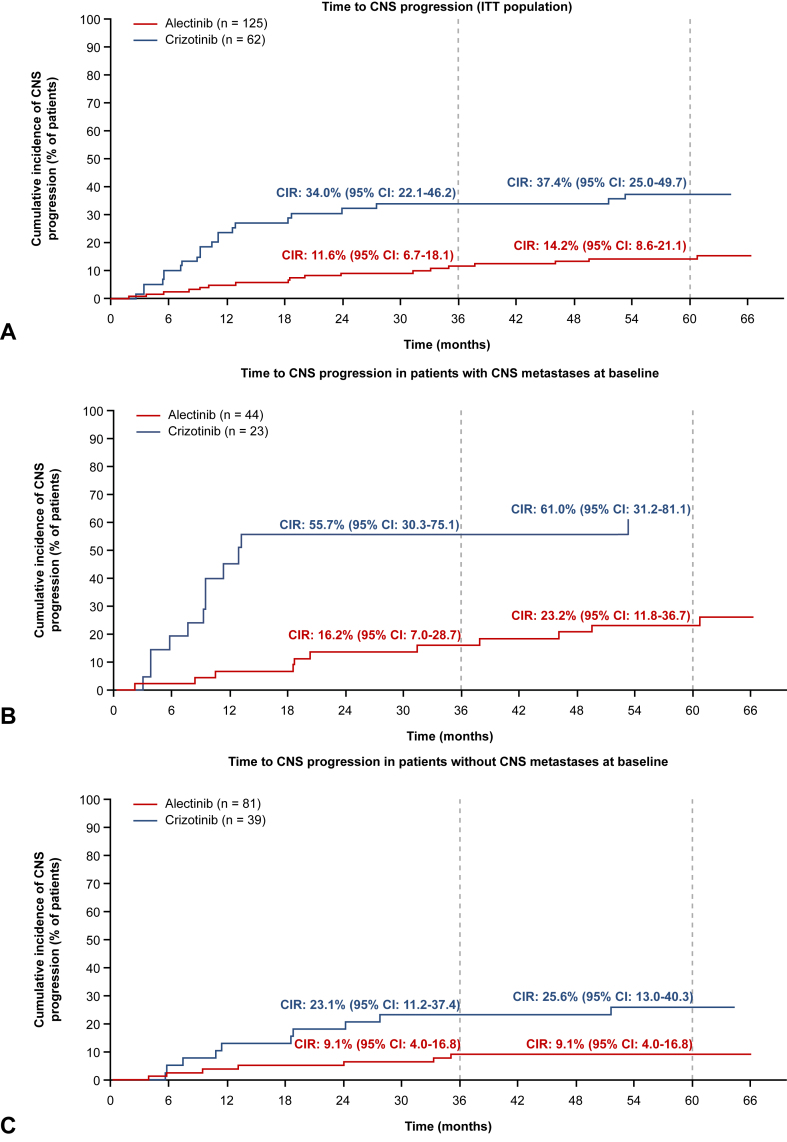
Time to CNS progression in *(A)* all patients, *(B)* patients with CNS metastases at baseline, and *(C)* patients without CNS metastases at baseline. CNS progression was derived from investigators’ assessment, defined as progression in target CNS lesions, the appearance of new CNS lesion(s) or unequivocal progression of non-target CNS lesion(s), according to Response Evaluation Criteria in Solid Tumors version 1.1. CI, confidence interval; CIR, cumulative incidence rate; CNS, central nervous system; ITT, intent-to-treat.

The investigator-assessed cumulative incidence rate (CIR) for CNS progression was 11.6% (95% CI: 6.7–18.1) in alectinib-treated patients and 34.0% (95% CI: 22.1–46.2) in crizotinib-treated patients at Month 36. This trend was observed across time points: at Month 60, the CIR for CNS progression was 14.2% (95% CI: 8.6–21.1) with alectinib versus 37.4% (95% CI: 25.0–49.7) with crizotinib (Fig. 3*A*). The cause-specific HR for CNS progression without prior systemic progression with alectinib versus crizotinib was 0.16 (95% CI: 0.08–0.32). In patients with baseline CNS metastases, the CIRs for CNS progression with alectinib versus crizotinib, respectively, were 16.2% (95% CI: 7.0–28.7) versus 55.7% (95% CI: 30.3–75.1) at Month 36, and 23.2% (95% CI: 11.8–36.7) versus 61.0% (95% CI: 31.2–81.1) at Month 60 (Fig. 3*B*). In patients without baseline CNS metastases, the CIRs for CNS progression at Month 36 were 9.1% (95% CI: 4.0–16.8) with alectinib versus 23.1% (95% CI: 11.2–37.4) with crizotinib, and 9.1% (95% CI: 4.0–16.8) versus 25.6% (95% CI: 13.0–40.3) at Month 60, respectively (Fig. 3*C*).

A lower proportion of subsequent anti-cancer therapies were reported after PD in the alectinib arm (42 of 68 [61.8%]) versus the crizotinib arm (38 of 48 [79.2%]) (Table 1). Overall, 36.8% (25 of 68) and 58.3% (28 of 48) of patients, treated with alectinib or crizotinib, respectively, received an ALK inhibitor following PD, most commonly alectinib, lorlatinib, or brigatinib. Furthermore, asymptomatic CNS PD was less common in patients receiving alectinib (13 of 125; 10.4%) than crizotinib (16 of 62; 23.8%), and the median duration of study drug treatment beyond isolated asymptomatic CNS PD was longer in the alectinib (11.2 mo, range: 0–39) than the crizotinib arm (3.9 mo, range: 0–34).

**Table 1:** Subsequent Anti-Cancer Therapy in Patients From the Alectinib Arm and the Crizotinib Arm, Following Disease Progression While on Study Treatment

Subsequent Anti-Cancer Therapy	Alectinib (n = 125)	Crizotinib (n = 62)
Patients with PD, n	68	48
Anti-cancer therapy after PD, n (%)	42 (61.8)	38 (79.2)
ALK inhibitor, n (%)	25 (36.8)	28 (58.3)
Alectinib	5 (7.4)	14 (29.2)
Lorlatinib	8 (11.8)	6 (12.5)
Brigatinib	6 (8.8)	7 (14.6)
Crizotinib	7 (10.3)	2 (4.2)
Ceritinib	3 (4.4)	4 (8.3)
Ensartinib	2 (2.9)	1 (2.1)
Investigational drug	1 (1.5)	0 (0.0)
Chemotherapy, n (%)	24 (35.3)	15 (31.3)
Anti-VEGF therapies, n (%)	9 (13.2)	3 (6.3)
Immunotherapy, n (%)	3 (4.4)	2 (4.2)
Other therapies, n (%)	6 (8.8)	7 (14.6)

PD, progressive disease; VEGF, vascular endothelial growth factor.

### Updated Safety Outcomes

The median duration of treatment at the updated data cutoff was 42.3 months in the alectinib versus 12.6 months in the crizotinib arm; 39.2% (49 of 125) of patients who received alectinib and 8.1% (five of 62) of patients who received crizotinib were still receiving study treatment at data cutoff.

Alectinib had a favorable safety profile relative to crizotinib, with fewer patients experiencing grade 3 to 5 AEs (48.0% versus 54.8%, respectively). The percentage of patients experiencing serious AEs was similar for alectinib and crizotinib (28.0% versus 29.0%, respectively), and patients experienced AEs leading to treatment discontinuation in both the alectinib arm (11.2%) and the crizotinib arm (14.5%) (Table 2). Compared with crizotinib, patients who received alectinib experienced fewer grade 3 to 5 treatment-related AEs (TRAEs) (21.6% versus 43.5%, respectively), serious TRAEs (4.8% versus 17.7%, respectively), and TRAEs leading to treatment discontinuation (4.8% versus 11.3%, respectively) (Table 2).

**Table 2:** Safety Overview

Patients With at Least One AE, n (%)	Alectinib (n = 125)	Crizotinib (n = 62)
AE	125 (100.0)	62 (100.0)
Treatment-related AE	123 (98.4)	61 (98.4)
Serious AE	35 (28.0)	18 (29.0)
Treatment-related serious AE	6 (4.8)	11 (17.7)
Grade 3-5 AE	60 (48.0)	34 (54.8)
Treatment-related grade 3-5 AE	27 (21.6)	27 (43.5)
Fatal AE	5 (4.0)	3 (4.8)
Fatal treatment-related AE	0 (0.0)	2 (3.2)
AE leading to treatment discontinuation	14 (11.2)	9 (14.5)
Treatment-related AEs leading to treatment discontinuation	6 (4.8)	7 (11.3)
AE leading to dose modification or interruption	52 (41.6)	25 (40.3)
Treatment-related AEs leading to dose modification or interruption	43 (34.4)	21 (33.9)
AE leading to dose reduction	33 (26.4)	17 (27.4)
Treatment-related AEs leading to dose reduction	30 (24.0)	16 (25.8)
AE leading to dose interruption	33 (26.4)	19 (30.6)
Treatment-related AEs leading to dose interruption	23 (18.4)	14 (22.6)

AE, adverse event.

Blood bilirubin increase (alectinib 57.6% versus crizotinib 4.8%), blood creatine phosphokinase increase (48.8% versus 30.6%), and anemia (47.2% versus 24.2%) were the most common AEs with greater than or equal to 10% difference in frequency between the treatment groups (Table 3).

**Table 3:** Adverse Events With Greater Than or Equal to 10% Difference in Frequency Between the Treatment Arms

Adverse Event, n (%)	Alectinib (n = 125)	Crizotinib (n = 62)
Blood bilirubin increased	72 (57.6)	3 (4.8)
Blood creatine phosphokinase increased	61 (48.8)	19 (30.6)
Anemia	59 (47.2)	15 (24.2)
Alanine aminotransferase increased	58 (46.4)	37 (59.7)
Constipation	50 (40.0)	34 (54.8)
Bilirubin conjugated increased	42 (33.6)	3 (4.8)
Sinus bradycardia	43 (34.4)	13 (21.0)
Weight increased	39 (31.2)	3 (4.8)
Blood alkaline phosphatase increased	34 (27.2)	9 (14.5)
Rash	25 (20.0)	5 (8.1)
Diarrhea	22 (17.6)	31 (50.0)
Blood thyroid-stimulating hormone increased	23 (18.4)	1 (1.6)
Hematuria	21 (16.8)	1 (1.6)
Nasopharyngitis	20 (16.0)	3 (4.8)
Blood bilirubin unconjugated increased	19 (15.2)	0 (0.0)
Dizziness	12 (9.6)	13 (21.0)
Decreased appetite	10 (8.0)	23 (37.1)
Vomiting	10 (8.0)	23 (37.1)
Nausea	9 (7.2)	21 (33.9)
White blood cell count decreased	8 (6.4)	19 (30.6)
ɣ-glutamyltransferase increased	9 (7.2)	17 (27.4)
Neutrophil count decreased	5 (4.0)	16 (25.8)
Edema peripheral	8 (6.4)	15 (24.2)
Headache	7 (5.6)	11 (17.7)
Vision blurred	2 (1.6)	9 (14.5)
Dysgeusia	1 (0.8)	9 (14.5)

Grade 3 to 5 AEs with the greatest difference in incidence rate between alectinib and crizotinib were neutrophil count decrease (0.0% versus 14.5%, respectively) and weight increase (8.8% versus 1.6%, respectively) (Supplementary Table 2). The grade 3 to 5 TRAEs with the largest difference in incidence rate between the alectinib and crizotinib arms were neutrophil count decrease (alectinib 0.0% versus crizotinib 14.5%) and alanine aminotransferase increase (alectinib 1.6% versus crizotinib 6.5%) (Supplementary Table 3). Three fatal AEs occurred after the primary analysis in the alectinib arm (two patients died of unknown causes and one patient died owing to coronavirus disease 2019 pneumonia); these were considered unrelated to the study treatment.

## Discussion

After at least 5 years of follow-up, investigator-assessed PFS in the ALESIA study was consistent with the primary analysis results.^10^ Investigator-assessed median PFS was prolonged with alectinib (41.6 mo, 95% CI: 33.1–58.9) versus crizotinib (11.1 mo, 95% CI: 9.1–18.4) (HR = 0.33, 95% CI: 0.23–0.49). These updated data support the clinical benefit observed with alectinib versus crizotinib in patients with advanced *ALK*-positive NSCLC in the randomized phase 3 ALEX and J-ALEX studies.^5^^,^^6^^,^^8^^,^^9^

Notably, PFS was improved with alectinib versus crizotinib in both patients with and without baseline CNS metastases, with a larger improvement observed in patients with baseline CNS disease. In addition, the cumulative incidence of CNS progression was consistently lower over time with alectinib compared with crizotinib, suggesting that alectinib helps to control and prevent CNS disease in this patient population. These results are consistent with the CNS-protective effects of alectinib as described previously in patients with *ALK*-positive NSCLC; in the ALEX study, the time to CNS progression was significantly longer with alectinib versus crizotinib (cause-specific HR = 0.16, 95% CI: 0.10–0.28, *p* < 0.001); 12% (18 of 152) of alectinib-treated patients and 45% (68 of 151) of crizotinib-treated patients had a CNS progression event) demonstrating that treatment with alectinib reduces the risk of CNS progression in patients with *ALK*-positive NSCLC.^11^

Median OS was NE in either treatment arm (alectinib arm: 95% CI: NE–NE; crizotinib arm: 95% CI: 45.5–NE) and the stratified OS HR (HR = 0.60, 95% CI: 0.37–0.99) was in line with findings from the 5-year OS analysis of ALEX (HR = 0.67, 95% CI: 0.46–0.98).^9^ A clinically meaningful difference in survival duration was observed, with a 5-year OS rate of 66.4% (95% CI: 57.9–74.9) with alectinib versus 56.1% (95% CI: 43.0–69.1) with crizotinib.

A lower proportion of patients with PD (including symptomatic deterioration) were reported to have received subsequent anti-cancer therapy after alectinib (61.8%) than after crizotinib (79.2%). As seen in the ALEX study, ALK inhibitors were the most common post-progression therapy in both arms of ALESIA, although a lower proportion of patients with PD received an ALK inhibitor following alectinib (36.8%) than following crizotinib (58.3%); however, this was not seen for those who received post-progression chemotherapy (alectinib arm: 35.3%; crizotinib arm: 31.3%).^9^ This difference may be due to there being limited approved and experimental ALK inhibitors for patients with PD following alectinib treatment versus crizotinib treatment, and due to missing data on subsequent anti-cancer therapy for patients who were lost to follow-up.

In this long-term safety update from ALESIA, a favorable safety profile for alectinib relative to crizotinib was observed, despite a three-times longer duration of alectinib treatment. The most frequently reported AEs in this updated analysis were generally consistent with the primary analysis.^10^ However, the rate of serious AEs in the alectinib arm increased from the primary analysis (28.0% versus 15.0%), which may be owing to the longer duration of alectinib treatment.^10^ Nevertheless, the newly reported serious AEs were consistent with the known safety profile of alectinib, and incidence rates of serious AEs were still comparable between arms (28.0% with alectinib versus 29.0% with crizotinib). Newly reported grade 3 to 5 AEs were consistent with the known safety profile of alectinib.

The limitations of this analysis include the open-label study design which may have biased the results, as previously discussed, although the primary analysis of ALESIA used IRC assessments to mitigate this.^10^ Due to the dosage difference between alectinib and crizotinib (with four capsules being required for alectinib versus one for crizotinib), an open-label study was necessary to prevent increasing patient and overall study burden. In addition, OS data remain immature.

Previous studies have investigated the efficacy and safety of other ALK inhibitors, such as ceritinib, brigatinib, and lorlatinib in patients with advanced *ALK*-positive NSCLC^12^, ^13^, ^14^; cross-study comparisons should be interpreted with caution, owing to differences in study design, and populations. In the ALTA-1 study,^13^ patients who received brigatinib had a higher investigator-assessed median PFS (30.8 mo, 95% CI: 21.3–40.6) than patients who received crizotinib (9.2 mo, 95% CI: 7.4–12.7); median OS was NR in either treatment arm. Compared with crizotinib, brigatinib reduced the risk of intracranial progression by 71% in patients who had baseline brain metastases (HR = 0.29, 95% CI: 0.17–0.51). Higher rates of grade ≥3 AEs, dose reductions, and discontinuations were reported in patients who received brigatinib versus crizotinib.^13^ In the phase 3 CROWN study,^14^ median investigator-assessed PFS were NR (95% CI: NR–NR) with lorlatinib versus 9.1 months (95% CI: 7.4–10.9) with crizotinib. Lorlatinib was associated with a longer time to intracranial progression than crizotinib in patients with baseline brain metastases (HR = 0.10, 95% CI: 0.04–0.27). Higher rates of grade 3 to 4 AEs were reported in the lorlatinib arm compared with the crizotinib arm (76% versus 57%, respectively); 7% and 10% of patients in these two arms discontinued treatment owing to AEs.^14^

However, updated data from this study are consistent with ALEX and confirm alectinib as a standard-of-care treatment option for advanced *ALK*-positive NSCLC.

## Conclusions

Updated efficacy and safety results from ALESIA after more than or equal to 5 years of follow-up are consistent with the primary analysis, and with data from the global ALEX study, supporting the finding that 600 mg twice-daily alectinib is effective and well tolerated as a first-line treatment for patients with advanced *ALK*-positive NSCLC.

The mature investigator-assessed PFS confirmed the systemic and CNS efficacy of alectinib versus crizotinib in Asian patients with previously untreated *ALK*-positive NSCLC. In addition, a clinically meaningful difference in OS was seen between the treatment arms in favor of alectinib, and a consistently lower CIR for CNS progression was also observed with alectinib versus crizotinib. Despite the longer duration of treatment in the alectinib versus the crizotinib arm, the updated safety data were still in favor of alectinib, and no new safety concerns were observed.

In conclusion, the clinical benefit observed after more than or equal to 5 years of follow-up, coupled with a tolerable and manageable safety profile, confirms alectinib as the standard-of-care treatment for patients with advanced *ALK*-positive NSCLC.

## CRediT Authorship Contribution Statement

**Caicun Zhou:** Investigation; Writing – Original draft preparation; Writing – Reviewing and editing.

**You Lu:** Investigation; Writing – Original draft preparation; Writing – Reviewing and editing.

**Sang-We Kim:** Investigation; Writing – Original draft preparation; Writing – Reviewing and editing.

**Thanyanan Reungwetwattana:** Investigation; Writing – Original draft preparation; Writing –Reviewing and editing.

**Jianying Zhou:** Investigation; Writing – Original draft preparation; Writing – Reviewing and editing.

**Yiping Zhang:** Investigation; Writing – Original draft preparation; Writing – Reviewing and editing.

**Jianxing He:** Investigation; Writing – Original draft preparation; Writing – Reviewing and editing.

**Jin-Ji Yang:** Investigation; Writing – Original draft preparation; Writing – Reviewing and editing.

**Ying Cheng:** Investigation; Writing – Original draft preparation; Writing – Reviewing and editing.

**Se-Hoon Lee:** Investigation; Writing – Original draft preparation; Writing – Reviewing and editing.

**Jianhua Chang:** Investigation; Writing – Original draft preparation; Writing – Reviewing and editing.

**Jian Fang:** Investigation; Writing – Original draft preparation; Writing – Reviewing and editing.

**Zhe Liu:** Investigation; Writing – Original draft preparation; Writing – Reviewing and editing.

**Lilian Bu:** Formal analysis; Validation; Writing – Original draft preparation; Writing – Reviewing and editing.

**Li Qian:** Formal analysis; Validation; Writing – Original draft preparation; Writing – Reviewing and editing.

**Tingting Xu:** Formal analysis; Methodology; Project administration; Writing – Original draft preparation; Writing – Reviewing and editing.

**Venice Archer:** Formal analysis; Methodology; Project administration; Writing – Original draft preparation; Writing – Reviewing and editing.

**Magalie Hilton:** Formal analysis; Validation; Visualization; Writing – Original draft preparation; Writing – Reviewing and editing.

**Mingzhu Zhou:** Formal analysis; Validation; Writing – Original draft preparation; Writing – Reviewing and editing.

**Li Zhang:** Investigation; Writing – Original draft preparation; Writing – Reviewing and editing.

## Disclosure

Prof. Caicun Zhou reports receiving consulting fees from Innovent Biologics, Jiangsu Hengrui Pharmaceuticals, Qilu Pharmaceutical, and TopAlliance Biosciences; and payment or honoraria for lectures, presentations, speakers bureaus, manuscript writing or educational events from Alice Pharma, AnHeart Therapeutics, Amoy Diagnostics, Boehringer Ingelheim, CStone Pharmaceuticals, Eli Lilly, F. Hoffmann-La Roche Ltd., Innovent Biologics, Jiangsu Hengrui Pharmaceuticals, Luye Pharma Group, Merck Sharp & Dohme, Qilu Pharmaceutical, Sanofi, and TopAlliance Biosciences. Prof. You Lu reports invited speaker roles for BeiGene, Roche/Genentech, AstraZeneca, Pfizer; advisory roles for Roche/Genentech, AstraZeneca, Pfizer, Bristol-Myers Squibb, Merck Sharp & Dohme, and BeiGene; and leadership roles for Roche/Genentech, AstraZeneca, and BeiGene. Dr. Sang-We Kim reports invited speaker roles for Amgen, Boehringer Ingelheim, Janssen, Novartis, and Takeda; receiving research funding from 10.13039/100019265Yuhan; and advisory roles from Amgen, AstraZeneca, Boehringer Ingelheim, Janssen, Novartis, Takeda, and Yuhan. Dr. Thanyanan Reungwetwattana reports advisory board roles from Amgen, AstraZeneca, Boehringer Ingelheim, Bristol-Myers Squibb, F. Hoffmann-La Roche Ltd., Johnson & Johnson, Merck Sharp & Dohme, Novartis, and Yuhan; participation in clinical research for AstraZeneca, Boehringer Ingelheim, F. Hoffmann-La Roche Ltd., Merck Sharp & Dohme, Novartis, and Yuhan; and speaker roles for Amgen, AstraZeneca, Boehringer Ingelheim, Bristol-Myers Squibb, F. Hoffmann-La Roche Ltd., Merck Sharp & Dohme, Novartis, Yuhan, and Zuellig Pharma. Prof. Jianying Zhou, Prof. Yiping Zhang, Prof. Jianxing He, Prof. Jin-Ji.Yang and Prof. Ying Cheng report no conflicts of interest. Prof. Se-Hoon Le reports receiving research funding (institution) from 10.13039/100004325AstraZeneca, 10.13039/100019703Lunit, and 10.13039/100004334Merck; and consulting/advisory roles from AstraZeneca, Bristol-Myers Squibb/Ono Pharmaceuticals, F. Hoffmann-La Roche Ltd., IMBdx, Janssen, Eli Lilly, Merck, Novartis, Pfizer, and Takeda. Prof. Jianhua Chang, Dr. Jian Fang and Prof. Zhe Liu report no conflicts of interest. Ms. Lilian Bu, Dr. Li Qian and Dr. Tingting Xu report employment with F. Hoffmann-La Roche Ltd. Dr. Venice Archer and Ms. Magalie Hilton report employment and stocks/shares with F. Hoffmann-La Roche Ltd. Dr. Mingzhu Zhou reports employment with F. Hoffmann-La Roche Ltd. Prof. Li Zhang reports consulting/advisory roles for AstraZeneca and Innovent Biologics; and receiving research funding (institution) from Akeso Biopharma, AstraZeneca, Bristol-Myers Squibb, Chia Tai Tianqing Pharmaceutical Group, Junshi Biosciences, and Qilu Pharmaceutical.
