# Supplementary material for: Alectinib Versus Crizotinib in Asian Patients With Treatment-Naïve Advanced ALK-Positive NSCLC: Five-Year Update From the Phase 3 ALESIA Study
Source: JTO Clin Res Rep. 2024 Jun 27;5(9):100700. doi: 10.1016/j.jtocrr.2024.100700 (PMC11399573; doi:10.1016/j.jtocrr.2024.100700)
Supplement: Supplementary Table 1-3 [file mmc1.docx]

# Supplemental Material

## Supplementary Table 1. Baseline patient characteristics

| **Characteristic** | **Alectinib**  **(n = 125)** | **Crizotinib**  **(n = 62)** |
| --- | --- | --- |
| Median age, years (range) | 51 (21–78) | 49 (28–83) |
| Gender, n (%)  Male  Female | 64 (51.2)  61 (48.8) | 34 (54.8)  28 (45.2) |
| ECOG PS, n (%)  0-1  2 | 121 (96.8)  4 (3.2) | 61 (98.4)  1 (1.6) |
| Disease stage, n (%)  IIIB  IV | 13 (10.4)  112 (89.6) | 4 (6.5)  58 (93.5) |
| Adenocarcinoma histology, n (%) | 117 (93.6) | 59 (96.7) |
| Smoking status, n (%)  Active smoker  Non smoker  Past smoker | 4 (3.2)  84 (67.2)  37 (29.6) | 3 (4.8)  45 (72.6)  14 (22.6) |
| Prior brain radiation, n (%) | 8 (6.4) | 5 (8.1) |
| Prior chemotherapy for localized disease, n (%) | 7 (5.6) | 9 (14.5) |
| CNS metastases by IRC, n (%) | 44 (35.2) | 23 (37.1) |
| CNS metastases by investigator, n (%) | 42 (33.6) | 20 (32.3) |

CNS, central nervous system; ECOG PS, Eastern Cooperative Oncology Group performance status; IRC, independent review committee.

## Supplementary Table 2. Grade 3-5 AEs with a difference in incidence rate ≥2% between treatment arms in the safety population

| **AE grade 3-5**, n (%)  ***≥2% difference between treatment arms*** | **Alectinib**  **(n = 125)** | **Crizotinib**  **(n = 62)** |
| --- | --- | --- |
| Weight increased | 11 (8.8) | 1 (1.6) |
| Blood creatine phosphokinase increased | 8 (6.4) | 2 (3.2) |
| Hyperglycemia | 3 (2.4) | 0 (0.0) |
| Hyperuricemia | 3 (2.4) | 0 (0.0) |
| Dyspnea | 3 (2.4) | 0 (0.0) |
| Death | 3 (2.4) | 0 (0.0) |
| Alanine aminotransferase increased | 3 (2.4) | 4 (6.5) |
| Nausea | 1 (0.8) | 3 (4.8) |
| Rash | 1 (0.8) | 2 (3.2) |
| Neutrophil count decreased | 0 (0.0) | 9 (14.5) |
| Electrocardiogram QT prolonged | 0 (0.0) | 3 (4.8) |
| White blood cell count decreased | 0 (0.0) | 3 (4.8) |
| Decreased appetite | 0 (0.0) | 3 (4.8) |
| Hyponatremia | 0 (0.0) | 3 (4.8) |
| Interstitial lung disease | 0 (0.0) | 3 (4.8) |
| Vomiting | 0 (0.0) | 3 (4.8) |
| Bradycardia | 0 (0.0) | 2 (3.2) |
| Hepatic function abnormal | 0 (0.0) | 2 (3.2) |

AE, adverse event.

## Supplementary Table 3. Grade 3-5 TRAEs with a difference in incidence rate ≥2% between treatment arms in the safety population

| **TRAE grade 3-5, n (%)**  ***≥2% difference between treatment arms*** | **Alectinib**  **(n = 125)** | **Crizotinib**  **(n = 62)** |
| --- | --- | --- |
| Weight increased | 6 (4.8) | 0 (0.0) |
| Anemia | 3 (2.4) | 0 (0.0) |
| Alanine aminotransferase increased | 2 (1.6) | 4 (6.5) |
| Neutrophil count decreased | 0 (0.0) | 9 (14.5) |
| White blood cell count decreased | 0 (0.0) | 3 (4.8) |
| Nausea | 0 (0.0) | 3 (4.8) |
| Vomiting | 0 (0.0) | 3 (4.8) |
| Interstitial lung disease | 0 (0.0) | 3 (4.8) |
| Electrocardiogram QT prolonged | 0 (0.0) | 2 (3.2) |
| Hepatic function abnormal | 0 (0.0) | 2 (3.2) |
| Decreased appetite | 0 (0.0) | 2 (3.2) |
| Bradycardia | 0 (0.0) | 2 (3.2) |

TRAE, treatment-related adverse event.
